# Time and Demand are Two Critical Dimensions of Immunometabolism: The Process of Macrophage Activation and the Pentose Phosphate Pathway

**DOI:** 10.3389/fimmu.2015.00164

**Published:** 2015-04-08

**Authors:** Csörsz Nagy, Arvand Haschemi

**Affiliations:** ^1^Department of Laboratory Medicine (KILM), Medical University of Vienna, Vienna, Austria

**Keywords:** immunometabolism, inflammation, macrophage activation, metabolic reprograming, primary carbohydrate metabolism, pentose phosphate pathway, sedoheptulose kinase, time and demand

## Abstract

A process is a function of time; in immunometabolism, this is reflected by the stepwise adaptation of metabolism to sustain the bio-energetic demand of an immune-response in its various states and shades. This perspective article starts by presenting an early attempt to investigate the physiology of inflammation, in order to illustrate one of the basic concepts of immunometabolism, wherein an adapted metabolism of infiltrating immune cells affects tissue function and inflammation. We then focus on the process of macrophage activation and aim to delineate the factor time within the current molecular context of metabolic-rewiring important for adapting primary carbohydrate metabolism. In the last section, we will provide information on how the pentose phosphate pathway may be of importance to provide both nucleotide precursors and redox-equivalents, and speculate how carbon-scrambling events in the non-oxidative pentose phosphate pathway might be regulated within cells by demand. We conclude that the adapted metabolism of inflammation is specific in respect to the effector-function and appears as a well-orchestrated event, dynamic by nature, and based on a functional interplay of signaling- and metabolic-pathways.

## Concepts of Immunometabolism

The first concepts of immunometabolism date back to the pre-genomic age of biomedical research. As early as 1912, Levene and Meyer used dog blood-derived leukocytes to directly demonstrate that hexoses are converted into two molecules, each containing a chain of three carbons (1). They also provided further evidence that hexoses are the source of lactate and assumed that this process accounts for “synthethic purposes by the leukocytes.” This period is widely recognized as the onset of modern biochemistry and furthermore of immunometabolism.

Immunometabolism is also tightly linked to research on cancer metabolism, especially with regard to the pioneering work of Otto Warburg, wherein he further developed the concept of *cellular physiology* (2). It was revealed that exudate leukocytes have high *aerobic glycolysis*, while respiration was very low and it was concluded then that white blood cells must have a *cancer metabolism* (3, 4). However, they differentiated immune cell- and cancer metabolism in that cancer cells use aerobic glycolysis to live, while aerobic glycolysis in white blood cells is a sign of *aging* or *dying off*. With this background, Walter Kempner and Ernst Peschel, both from the Bergmann’sche Institut at the Charite in Berlin, published their work with the German title: “Stoffwechsel der Entzündung” (Metabolism of Inflammation) (5). In 1930, they formulated two fundamental questions: what are the specific reactions of inflammation? Which processes lead to cell migration and subsequently to tissue swelling or necrosis? They presumed that an adapted cellular metabolism of white blood cells may play a major role in these processes. They tested their hypothesis in a human *in vivo* model of sterile-inflammation and provided fundamental new insights, which are still of relevance for today’s concepts of immunometabolism. Kempner and Peschel used the beetle-juice (cantharidin)-induced skin blister model and metabolically defined the inflamed human tissue in order to examine the *physiology of inflammation*. They observed a disrupted equilibrium of oxygen, CO_2_, sugar, lactate, and bicarbonate as a result of inflammation and concluded that this was induced by the metabolism found in infiltrating immune cells. They expected this to happen as a function of time. They demonstrated a drop in glucose over a period of 6–90 h and pulsed oral glucose administrations indicating that glucose replenishment from healthy tissue was also gradually declining. Within the inflamed area (the blister) also oxygen concentration declined. This was again attributed to high cellular respiration of infiltrated cells and a reduced gas-exchange with the healthy tissue. In addition to that, they measured a time-dependent increase in lactate and a decrease in the bicarbonate levels, which together could explain the decrease in pH of inflamed tissue, previously observed by Schade (6). Kempner and Peschel identified metabolic changes in inflamed tissue as a function of time, which is actively established by infiltrating “injured” immune cells with an adapted cellular metabolism (5). Thereby, they delineated a complex interplay between cellular metabolism and the physiology of inflammation. In 2011, the cantharidin-induced skin blister was re-evaluated and recommended as an excellent human *in vivo* model to study inflammation (7). This report also reveals that the infiltrating cells in this model are mainly neutrophils and monocytes/macrophages; these cells were probably also the cause for the observation by Kempner and Peschel.

Since then, a new school of immunobiology has started to reveal the molecular mechanism behind the observed metabolic-adaptation in various immune cells and models of immunology. As an example, the action of the pentose phosphate pathway (PPP) and the power of redox-biology, including superoxide production, were identified as essential in forming the respiratory-burst of phagocytes (8, 9). Also amino acid and lipid metabolism, as well as their adaptations, were characterized as fundamental to properly fuel the function of an immune response (10, 11). In recent years, however, new concepts in immunometabolism have evolved and further mechanistic-details have surfaced that enable us to better understand how these metabolic-adaptions are reached and regulated.

## Time Resolved Metabolic-Adaptations during Macrophage Activation

**Figure 1:**
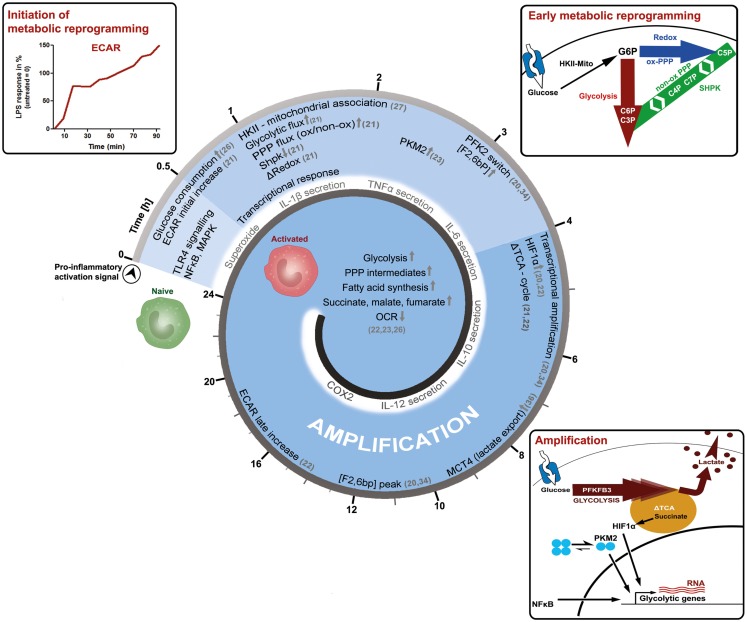
**Time-resolved metabolic reprograming during pro-inflammatory macrophage polarization**. This model illustrates the activation of a macrophage as a function of time and is based on the literature discussed in the main text. LPS-induced activation can be grouped into an initiation-, an early metabolic-reprograming,- and an amplification-phase. The initiation phase of the metabolic response is characterized by an increase in glucose consumption and in the extracellular acidification rate (ECAR). The early metabolic reprograming phase depicts the increase and rerouting of carbon flux through glycolysis and the PPP, events which also regulate the cellular redox-state. In this setting, the mitochondrial association of hexokinase-II (HKII) appears to provide sufficient levels of glucose 6-phosphate (G6P), while the downregulation of sedoheptulose kinase (Shpk, previously known as CARKL) appears to be necessary to maintain appropriate carbon flux at the interface of glycolysis and the PPP. During the amplification phase, this pro-glycolytic metabolic-phenotype is further strengthened. A switch toward the more active 6-phosphofructo-2-kinase (PFK2) enzyme PFKFB3 produces higher levels of fructose 2-bisphosphate [F2,6bP], thus allosterically activating PFK1 and enhancing glycolytic flux. Dimers of the pyruvate kinase M2 (PKM2), as well as accumulating succinate further augment metabolic reprograming by supporting HIF-1α dependent transcriptional induction of glycolytic genes. In the amplification phase, also the export of intracellular glycolysis-derived lactate through monocarboxylate transporter 4 (MCT4) becomes obligatory, which may otherwise inhibit PFK1. These initial events lead to more prominent metabolic changes observed 24 h after macrophages have encountered the pro-inflammatory stimuli. However, further time-resolved data is required to refine these processes and our current perspective, how cellular metabolism of macrophages adapts during activation.

Macrophages are important immune cells, which regulate tissue homeostasis by sensing and interpreting cell injury and infection, the classic triggers of an inflammatory response (12). Today, macrophages are classified according to the activation stimuli into at least two polarization states, the classic M1 (representing a pro-inflammatory phenotype) and the alternative M2 macrophage (representing an anti-inflammatory or homeostasis inducing phenotype), in order to discriminate between the effector phenotypes resulting from the distinct activation signals (13). However, *in vivo* macrophages rather appear to blend into various “shades of activation,” while retaining some of their plasticity (14–18). Furthermore, macrophage populations and phenotypes can dramatically change over time, as exemplified by the finding that the inflammatory response is a spatially and temporally coordinated process. Recently, the polarization process of macrophages has been further associated with the reprograming of cellular metabolism (19–25). Information processing by signal-transduction pathways starts shortly after activation and is temporally coordinated, reflected by the phosphorylation and de-phosphorylation of signal transducers and effector molecules. The question arises how the reprograming of primary carbohydrate metabolism is timed in the process of macrophage activation. We would like to present more detailed and more importantly time-resolved information on key events, which appear to establish a pro-inflammatory M1-like metabolic-phenotype induced by lipopolysaccharide (LPS, Figure 1).

After only 20 min of *in vitro* LPS-stimulation, simultaneously with prime signaling events, the glucose uptake of cells approximately doubles (26). At the same time, the extracellular acidification rate (ECAR), an indirect measure of aerobic glycolysis, also increases until reaching a certain plateau-state, to then adapt, and further increase (21). This response indicates that LPS leads to a rapid induction of glycolytic flux, which is modulated and amplified in multiple steps (Figure 1). The extension phase of ECAR is accompanied by a slow and marginal decrease in the oxygen consumption rate (OCR). The molecular mechanisms leading to these immediate early metabolic events, however, are not known and acidification may also result from sources other than the formation of lactic acid.

However, 1 h after LPS stimulation, the mRNA of the glucose transporter (GLUT1) is induced and the uptake of glucose further increases (26). After uptake of glucose, it becomes phosphorylated by hexokinases (HK) to glucose 6-phosphate (G6P), which can then be diverted into various catabolic and anabolic pathways. Non-stationary metabolic flux analysis, tracking the fate of intracellular glucose during macrophage activation, reveals that already 1 h after LPS-exposure a considerable amount of glucose is used by both, glycolysis and the PPP (21). In rat-Kupffer cells, which are specialized liver macrophages, as well as murine dendritic cells, HK-II was shown to associate with mitochondria within an hour after LPS stimulation (27, 28). A similar mechanism is observed in cancer cells, where mitochondrial matrix derived ATP is channeled to HK-II and thereby augmenting the glycolytic flux (29). Recently, the sedoheptulose kinase (Shpk, formerly known as CARKL*)* was characterized as a unique heptose kinase, phosphorylating sedoheptulose (a ketoheptose) to sedoheptulose 7-phosphate (S7P), which can then act as a reaction partner of glyceraldehyde 3-phosphate (G3P) in the non-oxidative PPP (21, 30–32). In macrophages, the mRNA of Shpk is rapidly down-regulated by LPS but not by interleukin (IL)-4 stimulation (21). Regulation of Shpk will be further discussed in the next section. Also, approximately after 1 h, LPS specifically induces pyruvate kinase M2 (PKM2) protein expression and phosphorylation, which becomes further augmented in the late phase of macrophage activation (23). Phosphorylation of PKM2 favors dimeric configuration and PKM2 translocation into the nucleus, where it acts together with hypoxia-inducible factor 1-alpha (HIF1α) as a transcriptional inducer of interleukin 1-beta (IL-1β) and more importantly of glycolytic genes like PFK, constituting an amplification loop in the intermediate and late phase of macrophage activation (23, 33). Within 2–4 h after activation by LPS, an isoform switchs from the liver-type 6-phosphofructo-2-kinase (PFKFB1 aka PFK2) to the ubiquitous and more active PFKFB3 occurs (34). This is also observed when LPS is used in combination with interferon gamma (IFNγ) to induce a pro-inflammatory macrophage activation (20). PFKFB3 produces augmented levels of fructose 2, 6-bisphosphate (F2,6bP), which then functions as an allosteric activator of 6-phosphofructo-1-kinase (PFK1) to further sustain the pro-glycolytic program (Figure 1). Interestingly in yeast, PFK1 derived F1,6bP allosterically activates PKM2, indicating the presence of metabolic feedback loops (35).

Approximately 4–6 h after macrophage activation, the export of glycolytic lactate appears to become mandatory for the activation process as indicated by the increased expression of monocarboxylate transporter 4 (MCT4) (36). Knockdown of MCT4 results in enhanced intracellular lactate accumulation, a decreased expression of LPS-induced glycolytic enzymes and an attenuated secretion of tumor necrosis factor-alpha (TNFα) and IL-6. Accumulating intracellular lactate might decrease glycolytic activity by inhibiting PFK1, an enzyme which may reach maximal activity in the later phase, as indicated by peaking F2,6bP concentrations and PFKFB3 mRNA levels at 6–12 h (34, 37). Also, approximately 4 h after initiation of macrophage polarization by LPS, the tricarboxylic acid (TCA) cycle changes its operational mode from a catabolic pathway to a partly anabolic system (21, 22). The TCA-cycle metabolite succinate accumulates in a macrophage cell line and bone marrow derived macrophages (BMDMs) (21, 22). Succinate, derived by glutamine-dependent anerplerosis and gamma-aminobutyric acid (GABA)-shunt, was shown to inhibit the prolyl hydroxylase-dependent degradation of HIF1α and to enhance IL-1β production (22). Increased succinate levels may also increase succinylation of metabolic enzymes such as glyceraldehyde 3-phosphate dehydrogenase (GAPDH), transaldolase (TALDO), and lactate dehydrogenase (LDH) A-chain, possibly further shaping late phase metabolic adaptations (22, 38). Succinate dependent HIF1α stabilization as well as increased succinylation are both suppressed by the inhibition of glycolysis, indicating that these processes are dependent on increased glycolytic flux (22, 23). Approximately 24 h after LPS-stimulation, metabolic reprograming is firmly established: glycolytic gene expression and metabolites are increased, as well as lactate and ECAR (22, 23). The TCA-cycle supports increased fatty acid synthesis as well as the formation of cycle intermediates (succinate, malate, fumarate), while OCR is reduced, indicating a significant decline in oxidative metabolism (22, 23).

To briefly summarize the overall consequences of the discussed adaptations: LPS stimulated macrophages increase aerobic glycolysis and PPP activity, reduce mitochondrial respiration, and reconfigure the TCA-cycle. In order to replenish NAD^+^ for glycolysis, lactate production and secretion are enhanced, leading to acidification of the environment. Such a pro-inflammatory metabolism is important for the generation of redox-equivalents as well as precursor molecules such as amino acids, lipids, and nucleotides, sustaining a burst in pro-inflammatory mediator production (39, 40). In reference to protein-signal-transduction leading to the observed metabolic adaptions, nuclear factor kappa-B (NFκB) and HIF1α are two well-characterized transcription factors, which increase the expression of glycolytic genes (41, 42). In contrast to an pro-inflammatory activation of macrophages, alternative activation (i.e., by IL-4) is associated with mitochondrial biogenesis as well as increased fatty acid oxidation and oxidative phosphorylation, primarily driven by lysosomal lipolysis of endocytosed lipoprotein particles (24, 43). In general, the M2 metabolic program mainly relies on STAT6, PPARγ, and its co-activator PGC1β to promote oxidative metabolism. The manifold metabolic changes during macrophage activation as well as their regulatory mechanisms have been recently discussed in detail in some excellent reviews (44–46).

## The PPP Sustains the Metabolic Demand of Macrophages during Polarization

The PPP represents a prime example on how increased carbon-flux can contribute to mount the specific effector functions of LPS-activated macrophages by complementing their appropriate demands through supplying both redox-power and ribose moieties either at the same time or independently from each other. The PPP is divided into the oxidative (oxPPP) and non-oxidative branch (non-oxPPP). Briefly, the oxPPP, with glucose 6-phosphate dehydrogenase (G6PD) as its rate-limiting enzyme, is highly active in macrophages (21, 47), decarboxylates G6P, and forms ribose 5-phosphate (R5P) through three irreversible reactions, while simultaneously reducing two molecules of NADP^+^ to NADPH and liberating one molecule of CO_2_. The non-ox PPP consists of reversible reactions, which can either recycle R5P to glycolytic intermediates or use the latter to generate pentose phosphates (C5P) through reverse flux. The general aspects of PPP architecture and function have been reviewed elsewhere in great detail (48–50).

Oxidative PPP derived NADPH serves as a cofactor for NADPH-oxidase dependent reactive oxygen species (ROS) production, while also reducing oxidized redox-couples to simultaneously sustain an anti-oxidant response (i.e., glutathione and thioredoxin systems), thereby partly controlling the redox balance during macrophage activation (51, 52). Also, the function of many redox-sensitive signaling proteins, which are associated with the process of activation, are potentially dependent on increased flux through the oxPPP (53–59). Furthermore, NADPH is also critical for reductive biosynthesis serving activation associated membrane expansion and the production of lipid mediators such as prostaglandins. Remarkably, it was reported that NADPH levels undergo periodic oscillations in macrophages and neutrophils, which are tightly linked to superoxide oscillations and adapt, upon LPS stimulations to a higher frequency (60–63). These oscillations may depend on periodic glucose influx and PPP activity, and appear to encode information in their amplitude and frequency (64, 65). Severe G6PD deficient leukocytes have been associated with impairments in their oxidative burst, their bactericidal activity (66–68), their resistance to oxidative stress (69), as well as modified cytokine responses (70–72). Overexpression of G6PD in a macrophage cell line enhanced the activation of NFκB and p38-MAPK signaling pathways and potentiated the expression of pro-inflammatory cytokines as well as ROS production (73). In contrast to IL-1β, the production of TNFα and IL-6 does not appear to be directly dependent on aerobic glycolysis, as recently suggested by the activation of PKM2 and 2-deoxyglucose (2-DG) treatment, respectively (22, 23). Notably, 2-DG is a glycolytic inhibitor downstream from hexokinase and can therefore become phosphorylated to 2-deoxyglucose 6-phosphate, which is partly metabolized by the oxPPP in red blood cells (74). Whether this also occurs in macrophages or not remains to be investigated. Inhibition of G6PD or LDH, however, was shown to decrease TNFα and IL-6 levels, implicating that these cytokines are rather regulated by redox-state than simply by the increased glycolytic-flux (21). Apart from redox-power, the macrophage activation process also demands a large amount of pentose phosphates probably to sustain *de novo* nucleotide synthesis for their characteristic transcriptional response. In contrast to M2, M1 macrophages drastically change their transcriptional profile (40). Isotope distribution analysis of a non-stationary metabolic flux experiment with asymmetrically labeled glucose after 1 h of LPS-induced macrophage activation indicated that both ox- and non-oxPPP flux rates increase, while most of the pentose phosphates (C5) are derived from the non-ox branch (21).

**Figure 2:**
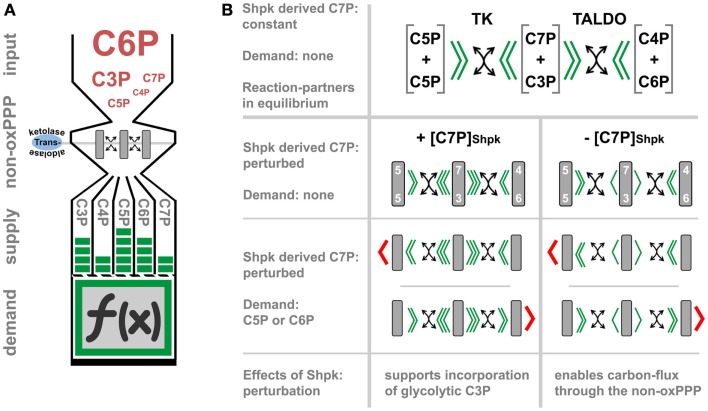
**The function and regulation of the non-oxidative PPP**. **(A)** represents a simplified model, which illustrates how transketolase (TK) and transaldolase (TALDO) may interconvert carbohydrate-phosphates of three to seven carbon-atoms in length (C3P to C7P) without the need of energy (carbon-scrambling) to account for the cellular demand, which in part defines cell function (indicated by the symbol *f* (*_x_*)). In **(B)**, we theoretically evaluate the regulatory effect of Shpk-derived sedoheptulose 7-phosphate ([C7P]_Shpk_) on non-oxPPP flux in the presence of TK and TALDO. Flux through the non-oxPPP, by its reversible reactions, is dependent on the stoichiometry of the reactants (indicated by green arrowheads). In contrast to TK- and TALDO-derived S7P, the phosphorylation of free sedoheptulose to S7P by Shpk requires energy in form of ATP. Assuming a constant contribution by Shpk, this additional source of S7P may therefore act as a thermodynamic buffer, which can be actively regulated to induce a non-equilibrium. In theory, perturbation of Shpk can either increase the resistance (increased [C7P]_Shpk_) or lower it (decreased [C7P]_Shpk_) to support shunting through the non-oxPPP. However, in the presence of TK or TALDO, an increased [C7P]_Shpk_ will promote the incorporation of glycolytic-G3P into the PPP. This model further illustrates that non-oxPPP flux-direction is also dependent on the demand of respective molecules (indicated by red arrowheads).

The non-oxPPP relies on transketolase (TK) and TALDO catalyzed reversible transfer of keto-groups to various aldose acceptors. TK uses thiamine pyrophosphate as cofactor to transfer two carbon (C2)-units, while TALDO can transfer C3-units by forming Schiff base intermediates (75, 76). Thereby, this pathway interconverts carbohydrate-phosphates of different chain length (C3P to C7P), without the need of energy in form of ATP (carbon scrambling, Figure 2A). The regulation of non-oxPPP is complex due to its reversible nature and still not fully understood. The flux-rate and its direction are generally thought to depend on thermodynamics, which impose a major constraint on the structure of metabolic pathways (77). However, the recent identification of Shpk indicates additional regulatory mechanism, which was previously not considered (Figure 2B) (78). In contrast to TK or TALDO, Shpk is reported to be regulated differently during LPS- and IL-4 induced polarization (21). LPS stimulation leads to a rapid down-regulation of Shpk mRNA in the early phase of macrophage activation in mice and humans likewise and *in vitro* as well as *in vivo*. In contrast to LPS, IL-4 stimulation maintains or even slightly increases Shpk levels (21). Counterbalancing LPS-induced down-regulation of Shpk by overexpression in a macrophage cell line resulted in an accumulation of pentose phosphates and an imbalance of the cellular redox system, as indicated by the accumulation of oxidized redox couples as well as blunted LPS-induced intracellular superoxide production (21). In theory, Shpk, by the formation of rate-limiting S7P, should increase the shunting of glycolysis-derived G3P into the non-oxPPP (78) and regulate oxPPP activity through the formation or recycling of pentose phosphates (79). So far, we have no confirmed mode-of-action, how Shpk activity actually regulates carbon-flux through the non-oxPPP, and no information on its activity and local distribution during macrophage activation. Therefore, we can only speculate on the consequences of Shpk regulation for the process of metabolic-adaptation (Figure 2B). Shpk-derived S7P may act as a thermodynamic buffer to support a stable non-equilibrium, which drives (low S7P) or inhibits (high S7P) carbon-flux through the non-oxPPP. However, flux-direction seems to be determined by demand and by the presence of TK and TALDO (Figure 2B). In addition to that, high S7P levels can directly modulate glycolytic flux through the inhibition of hexose phosphate isomerase, as well as by competitively inhibiting fructose 6-phosphate (F6P) phosphorylation by PFK (80, 81). Therefore, the consequences of Shpk regulation appear as strictly context dependent, which is defined by the demand of metabolites (i.e., C5P) and the presence or absence of other enzymes. We know that Shpk only partially colocalizes with G6PD in the cytoplasm of cells, which points out that there are instances where the ox- and the non-oxPPP are coupled to and uncoupled from each other (21). Information on the function of TK and TALDO in the process of macrophage activation is rare; however, both enzymes were tightly linked to oxidative stress-defense in other cell types (82–84). Notably, yeast seems to lack a Shpk homolog but utilizes a specific sedoheptulose–bisphosphatase [dephosphorylates sedoheptulose 1,7-bisphosphate (S1,7bP) to S7P] for riboneogenesis when the demand for nucleotide precursors is high (85). S1,7bP was previously reported to also exists in rat liver tissue (86, 87); however, there appear to be some major differences in the architecture of heptose metabolism (heptolysis) between fungi and vertebrates (78, 85).

In summary, these findings indicate that during macrophage activation the cellular demands are covered by a precisely coordinated interplay of many pathways to sustain such profound polarization events. The PPP appears as a versatile hub to reroute carbon moieties within the network of primary carbohydrate metabolism while independently controlling cellular redox-states.

## Conclusion

This collection of findings may support our perspective that time and demands are critical to understand the molecular events important to mount an immune response. Immunometabolism demonstrates its consequences for physiology at various levels including cells, tissues, organisms, and entire populations, as we currently experience with diseases like cancer, cardiovascular diseases, obesity, and diabetes to name but a few. Already, Kempner and Peschel considered diabetic patients in their investigations and noted a sustained glucose supply together with a prolonged inflammatory response compared to non-diabetics. Since then, many excellent studies further delineated the complex interplay of metabolism, the immune system and tissue function, and malfunction. At the molecular level, macrophages adapt their metabolism very early in the polarization process, which then become amplified over time. This highlights that we need to strongly consider the process leading to activation and not only the phenotypic “endpoints.” A macrophage located within a complex tissue microenvironment may go through multiple, subsequently occurring, activation events, which then may amplify or antagonize each other. It will be interesting to test *in vivo* if, and importantly how, subsequent or parallel cross-presentation of multiple activation-stimuli (i.e., pro- and anti-inflammatory signals such as LPS, IFNγ, TNFα, IL-6, IL-4, or IL-10) may skew and define the process of metabolic reprograming in macrophages.

## Conflict of Interest Statement

The authors declare that the research was conducted in the absence of any commercial or financial relationships that could be construed as a potential conflict of interest.
